# Corrective osteotomies of malunited proximal humerus fractures with 3D patient-specific guides: a feasibility study

**DOI:** 10.1016/j.jseint.2026.101784

**Published:** 2026-08-14

**Authors:** Stijn R.J. Mennes, Anne M.L. Meesters, Michel P.J. van den Bekerom, Ruurd L. Jaarsma, Job N. Doornberg, Frank F.A. IJpma, Nick Assink

**Affiliations:** a Department of Orthopaedic & Trauma Surgery, Flinders Medical Centre and Flinders University, Adelaide, Australia; b Shoulder and Elbow Unit, Department of Orthopaedic Surgery, OLVG, Amsterdam, The Netherlands; c Faculty of Behavioural and Movement Sciences, Department of Human Movement Sciences, Vrije Universiteit Amsterdam, Amsterdam Movement Sciences, Amsterdam, The Netherlands; d Department of Trauma Surgery, University Medical Center Groningen, Groningen, The Netherlands; e 3D Lab, University Medical Center Groningen, Groningen, The Netherlands; f Department of Orthopaedic Surgery, University Medical Center Groningen, Groningen, The Netherlands

**Keywords:** 3D technology, Patient-specific surgical guides, Corrective osteotomy, Proximal humerus fracture, Malunion, Virtual surgical planning

## Abstract

**Background:**

Corrective osteotomies for malunions after proximal humerus fractures are reserved for selected cases. These procedures are technically demanding, and surgical planning based on two-dimensional imaging, with free-hand operative correction, may lead to surgical inaccuracy. Three-dimensional (3D) pre-operative planning combined with patient-specific surgical guides and surgical simulation can overcome this issue. This study introduces 3D patient-specific guides for corrective osteotomies in proximal humeral malunions and determines the accuracy of achieved correction in human cadaveric shoulders with 2 innovative surgical techniques.

**Methods:**

Seven reversed corrective osteotomies were planned and performed on 4 Thiel-embalmed human specimens using 7 different respective 3D patient-specific cutting and reduction guides. Computed tomography scans were used to create 3D segmentations of all specimens. Subsequently, virtual surgical plans of osteotomies were created, and 3D-printed patient-specific cutting and reposition guides were designed and produced. Three humeri underwent an “extracapsular” corrective osteotomy, and 4 humeri underwent correction using a novel “humeral head-tuberosity separation” technique. Accuracy was assessed for valgus angulation and rotation by comparing pre-operative planning with post-operative results quantified with computed tomography scans.

**Results:**

The overall median difference between pre-operative planning and post-operative results was 5.2° (min-max: 2.9°-8.5°) for rotation and 3.4° (min-max: 2.4°-5.8°) for valgus angulation. For the “extracapsular” method, the median difference was 5.2° (min-max: 2.9°-7.0°) for rotation and 3.9° (min-max: 3.2°-5.8°) for valgus angulation. The median differences in rotation and valgus angulation for the “humeral head-tuberosity separation” corrections were 6.3° (min-max: 3.6°-8.5°) and 3.1° (min-max: 2.4°-3.6°), respectively.

**Conclusion:**

The application of 3D surgical planning and patient-specific guides is feasible for corrective osteotomies of malunited proximal humerus and resulted in reconstructions with less than 10° deviation from pre-operative planning, demonstrating acceptable technical accuracy and supporting the potential of this approach for future clinical application. The extracapsular osteotomy technique may serve as a solution for malunions with an intact anatomic relationship between the humeral head and greater tuberosity, whereas the novel osteotomy may be a solution for severe malunions with disrupted anatomy between the humeral head, greater tuberosity, and surgical neck. Future studies need to evaluate the use of the proposed techniques in patients with actual proximal humeral malunions.

## Background

**Figure 1:**
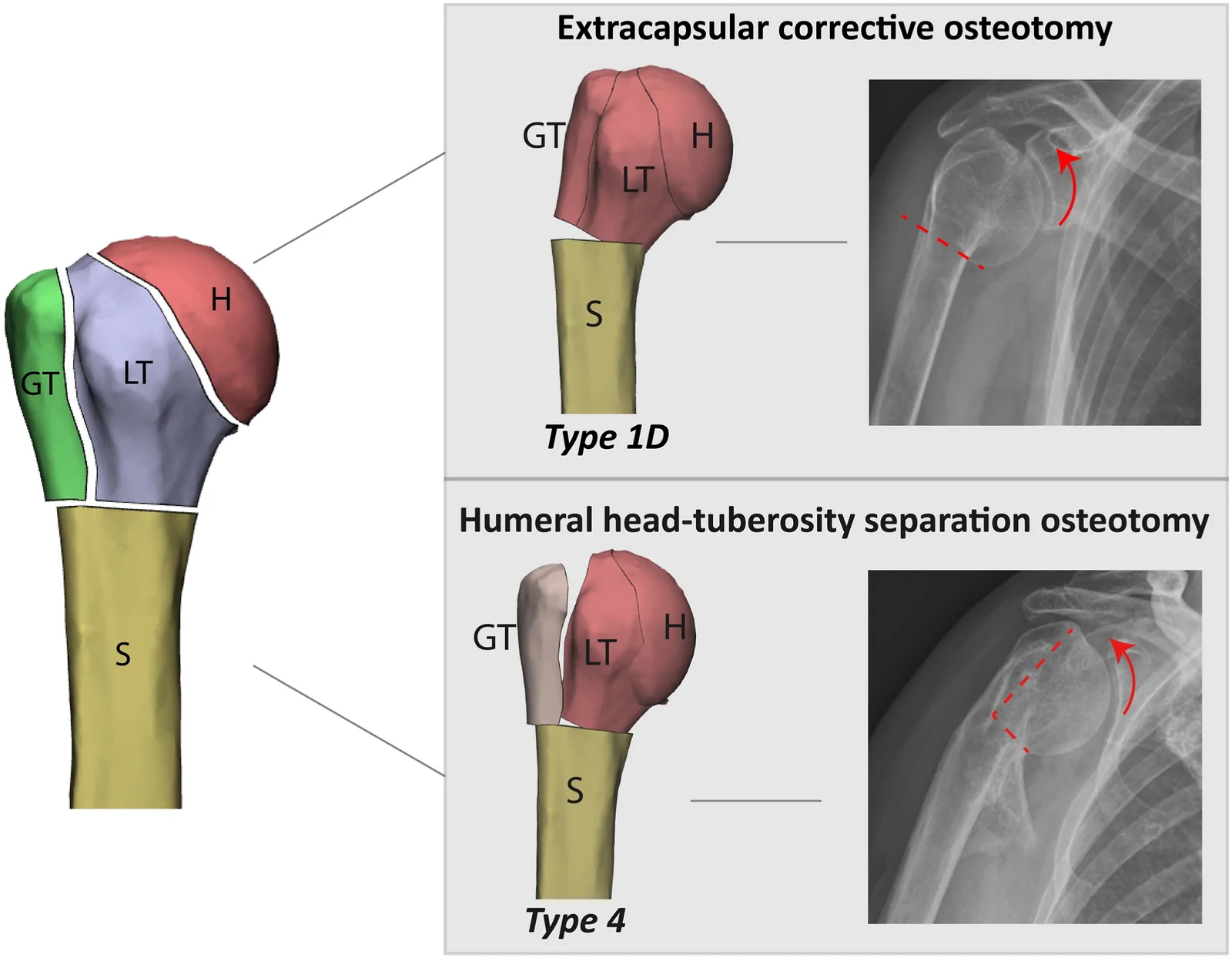
Schematic overview of the 2 described techniques. The *Upper* part represents the “extracapsular” technique for 1D (varus) deformities. The *Lower* part represents the “head-tuberosity separation” technique for type 4 (migrated GT) deformities. The red dashed lines represent the respective cuts, and the arrows represent the direction of the repositions. *GT*, greater tuberosity; *LT*, lesser tuberosity; *H*, humeral head; *S*, humeral shaft.

Proximal humerus fractures (PHFs) are among the most common fractures and comprise up to 10% of all fractures.^11^^,^^26^ Malunion, or bone healing in a non-anatomic position, is observed in 4%-20% of all fractures.^7^ While most malunited PHFs do not have clinical consequences, some may lead to pain and disability. Pain, stiffness, and limited active abduction or forward flexion are reported, especially in malunions with valgus or varus deformation and superior migration of the greater tuberosity (GT) resulting in sub-acromial impingement.^3^ Moreover, incongruity of the glenohumeral joint or soft-tissue alterations, such as capsular contractures due to scarring or rotator cuff deficiency, contribute to shoulder impairment.^1^^,^^4^ It is a result of either an initial reduction problem or a secondary displacement after fixation, or a secondary displacement due to loss of stability,^7^ and occurs after both non-operative and surgical treatment.^9^ Boileau et al classified these malunited fractures as type 1C (valgus), 1D (varus), and type 4 (GT deformity), respectively (Fig. 1).^5^^,^^15^ Surgical management is indicated in malunions resulting in either pain or restriction of shoulder movement.^21^ For type 1 fracture sequelae, arthroplasty may offer relatively acceptable and predictable functional outcomes.^15^ However, in sequelae with GT malunions or a requirement of a tuberosity osteotomy (type 4 malunions), outcomes are worse and less predictable, and complication rates increase.^1^^,^^5^ Moreover, arthroplasty may be less desirable for younger patients with malunions since this treatment option is generally reserved for the elderly.^13^

A commonly described treatment for malunions is a corrective osteotomy. While this procedure is frequently used in other long-bone deformities, it remains reserved for selected cases after PHFs. Although evidence is limited to few case series and small retrospective clinical studies, corrective osteotomies for malunions after PHFs have shown favorable results.^3^^,^^4^^,^^20^^,^^22^ Benegas et al^3^ and Solonen and Vastamaki^22^ describe the use of a valgus wedge osteotomy to correct varus malalignment, planned on radiographs, whereas Beredjiklian et al^4^ reported the use of isolated GT osteotomies to restore anatomy. Russo et al describe a more advanced technique with biplane or triplane osteotomies through the surgical neck and tuberosities, with the use of computed tomography (CT).^20^ However, this type of surgery is technically demanding because of unique, multi-planar deformities. It requires extensive pre-operative planning and is usually prepared using two-dimensional (2D) CT images or three-dimensional (3D) reconstruction without surgical simulation during pre-operative planning, leading to surgical inaccuracy and thereby hindering its widespread implementation.^10^ Corrective osteotomies using more advanced 3D pre-operative planning with surgical simulation and patient-specific surgical guides have been proven reproducible in other body regions, such as the distal radius and tibia.^14^^,^^16^ A theoretical guideline on 3D corrective osteotomies after PHFs has been written^24^; however, to our knowledge, the surgical accuracy of 3D patient-specific guides has never been evaluated in vitro nor in vivo.

This feasibility study aimed to explore the use of 3D patient-specific guides for corrective osteotomies after proximal humeral malunions and determine the accuracy of achieved correction in human cadaveric shoulders. We aimed to introduce and compare 2 distinctive 3D techniques for performing corrective osteotomies, each potentially suited to different malunion patterns. This study tested feasibility and determined post-operative accuracy of 1) an extracapsular osteotomy suitable for varus (type 1D) malunions, and 2) a humeral head-tuberosity separation osteotomy suitable for malunions with GT deformities (type 4).

## Methods

### Study setup

This feasibility study was performed using 4 Thiel-embalmed human specimens (2 male and 2 female) with 7 suitable proximal humeri without a prior injury to the bone. One humerus was excluded from the study because of a pre-existing bone defect to the proximal humeral shaft. The focus was on evaluating the feasibility and *accuracy of the achieved correction*. The correction was considered feasible if it could be performed through a deltopectoral approach without compromising essential anatomic structures, including the sub-scapularis tendon insertion on the lesser tuberosity; the supraspinatus, infraspinatus, and teres minor tendon insertions on the GT; and the long head of the biceps tendon. Three humeri underwent an “extracapsular” corrective osteotomy, and 4 humeri underwent correction with a novel “humeral head-tuberosity separation” technique using 3D surgical guides. Both the “extracapsular” and “humeral head-tuberosity separation” techniques were assessed to cover most commonly reported malunion types as shown in Figure 1. The osteotomies modified the humeral anatomy in the coronal and axial planes. In this study, the sagittal plane was not considered. All osteotomies were performed by orthopedic surgeons who fully completed their residency program. The humeri undergoing “extra-capsular” corrective osteotomies were female specimens, and the humeri undergoing “humeral head-tuberosity separation” osteotomies were male specimens.

In our experimental setup, a traditional approach using corrective osteotomies to return to an anatomic state was impossible since all specimens lacked deformities. Therefore, an alternative reversed corrective osteotomy was performed to evaluate the accuracy of 3D-assisted corrective osteotomies through the 2 different approaches. Locking plates (PHILOS, Synthes, Oberdorf, Switzerland) and small-fragment locking compression plate (Synthes, Oberdorf, Switzerland) were used as implants for fixation.

#### Pre-operative 3D virtual planning

From all specimens, a CT scan was obtained according to our standard clinical imaging protocol (0.6 mm slice thickness, voxel size 0.4 mm). The CT data were loaded into Mimics Medical software (version 27.0, Materialize, Leuven, Belgium) and segmented (based on the Hounsfield Units of bone) to generate 3D models of all humeri. These virtual 3D models were then imported into 3-Matic software (version 19.0, Materialize, Leuven, Belgium) to create a virtual surgical plan of the osteotomy and then design and produce the 3D-printed patient-specific cutting and reposition guides. All guides were designed to fit the underlying humerus and enable a patient-specific osteotomy with subsequent (reversed) correction based on our predefined angulation and rotation. Surgical guides were then manufactured from polyamide 12 using selective-laser-sintering additive manufacturing technology.

#### Surgical procedure—extracapsular osteotomy

**Figure 2:**
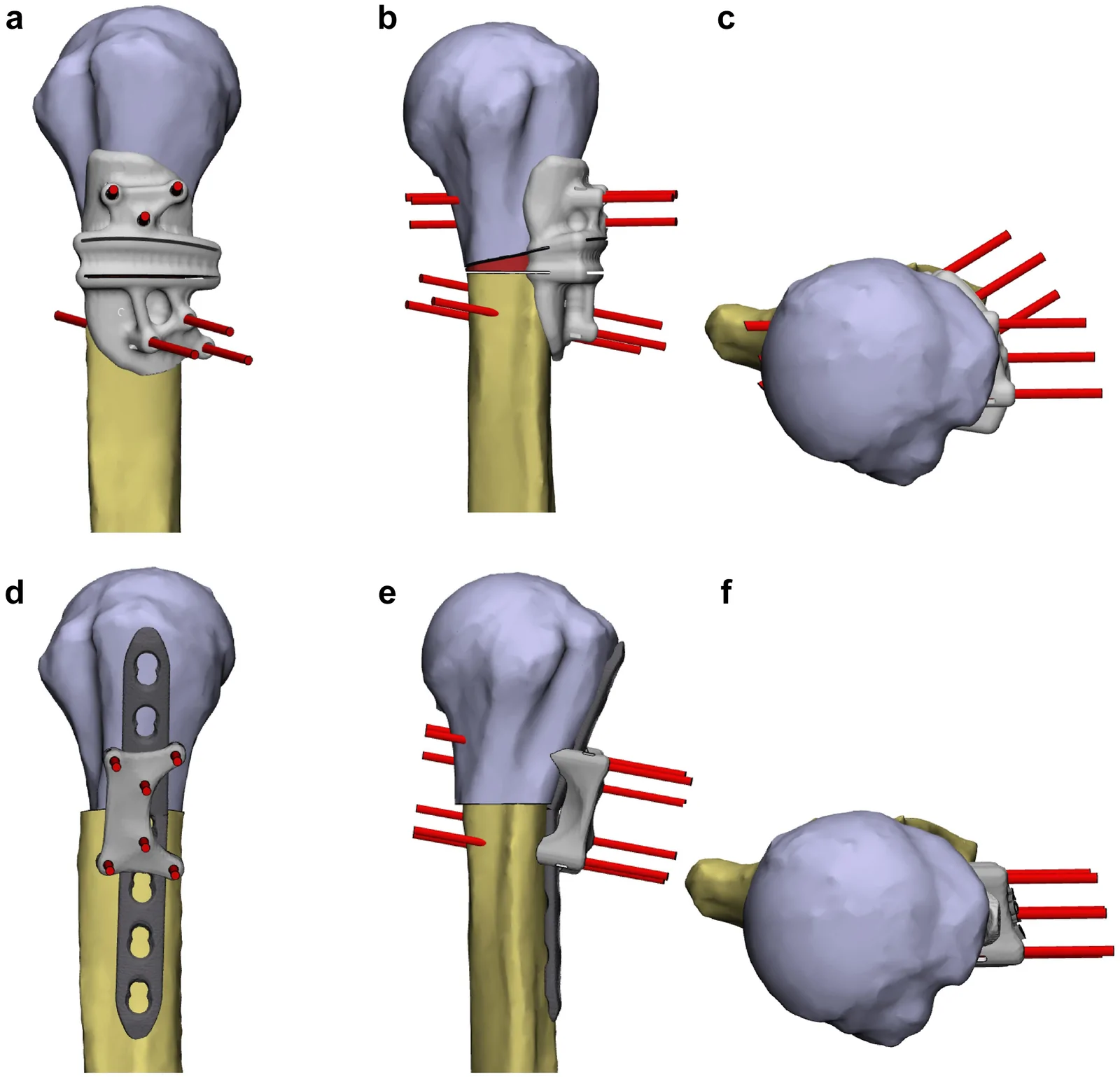
Three-dimensional planned corrective osteotomy according to the “extracapsular” method. (**a** and **b**) The patient-specific cutting guide, aligned to the humerus with K-wires, for an osteotomy through the humeral metaphysis. (**d** and **e**) The reduction guide, guided by K-wires, with the pre-bent plate to fixate the osteotomized proximal humerus. (**c** and **f**) Cranial views to illustrate the rotational difference.

**Figure 3:**
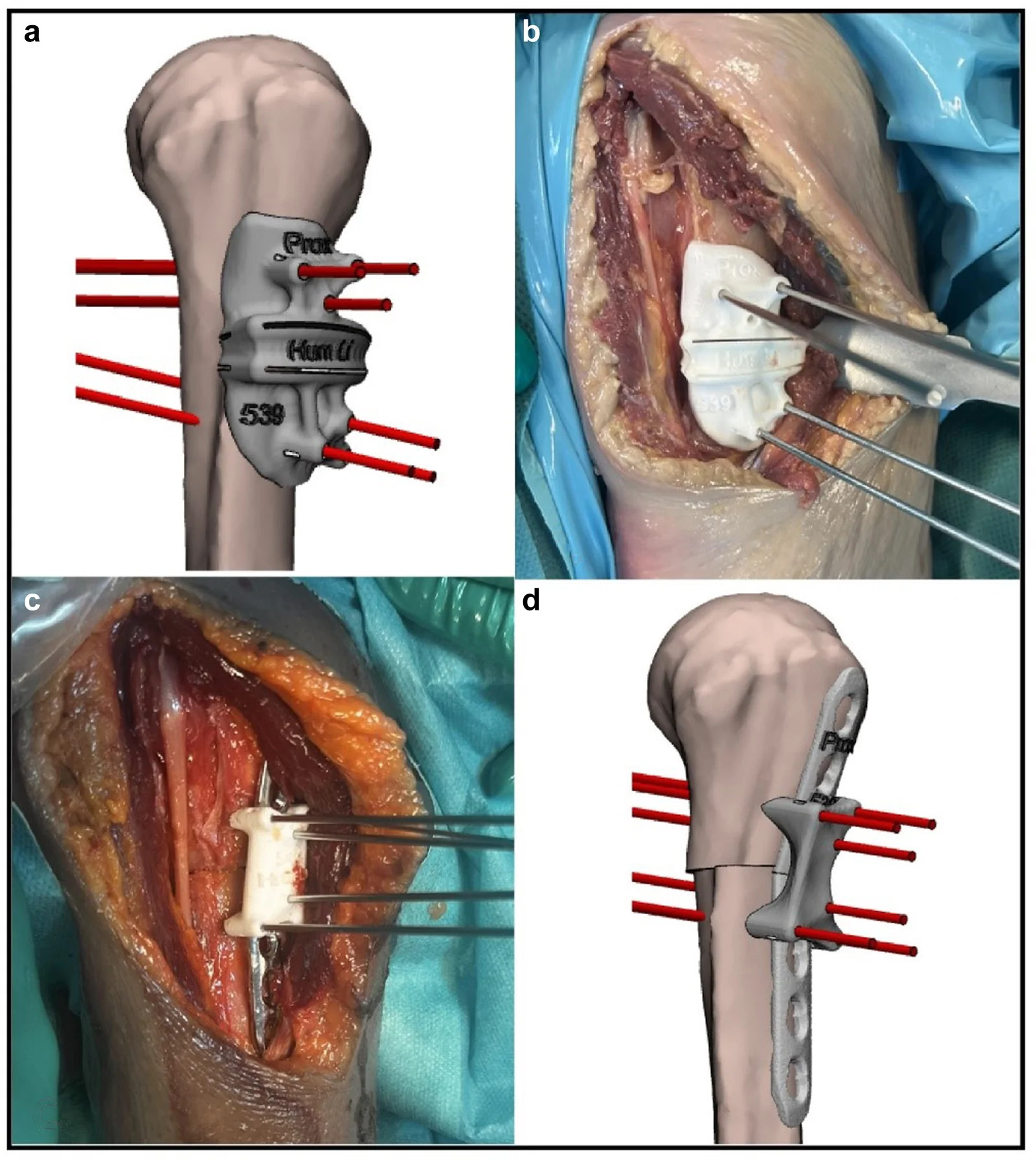
Visual representation of the “extracapsular” procedure. (**a** and **b**) The 3D re-constructed patient-specific cutting guide and the intra-operative example, respectively. (**c** and **d**) The intra-operative example and 3D re-constructed patient-specific reposition guide with the pre-bent small-fragment locking compression plate for fixation.

The first technique encompassed an extracapsular corrective osteotomy where Kirschner (K)-wires were placed first, followed by an osteotomy through the humeral metaphysis (using a patient-specific cutting guide) after which the humeral shaft was moved to 30° of internal rotation and 10° in valgus angulation (Fig. 2). A deltopectoral approach to the anterior proximal humerus was performed. First, after the preparation of the proximal humerus, the cutting guide (Figs. 2, *A*-*C*, 3, *A* and *B*) was aligned with the bone and secured on the bone using K-wires (Figs. 2, *A*-*C*, 3, *A* and *B*). Two cuts were performed using a cutting saw (max 0.6 mm thick), resulting in a wedge (Fig. 2, *B*). Subsequently the cutting guide was removed while leaving the K-wires in place, and the reposition guide was placed on the K-wires forcing the bone fragments into the desired pre-planned position (Figs. 2, *D-F*, 3, *C* and *D*). A small-fragment locking compression plate (Synthes) was bent on a 3D-printed model of the planned outcome and placed on the osteotomized proximal humerus to fixate the achieved position (Figs. 2, *D-F*, and 3, *C* and *D*). Finally, the reposition guide was removed.

### Surgical procedure—humeral head-tuberosity separation osteotomy

**Figure 4:**
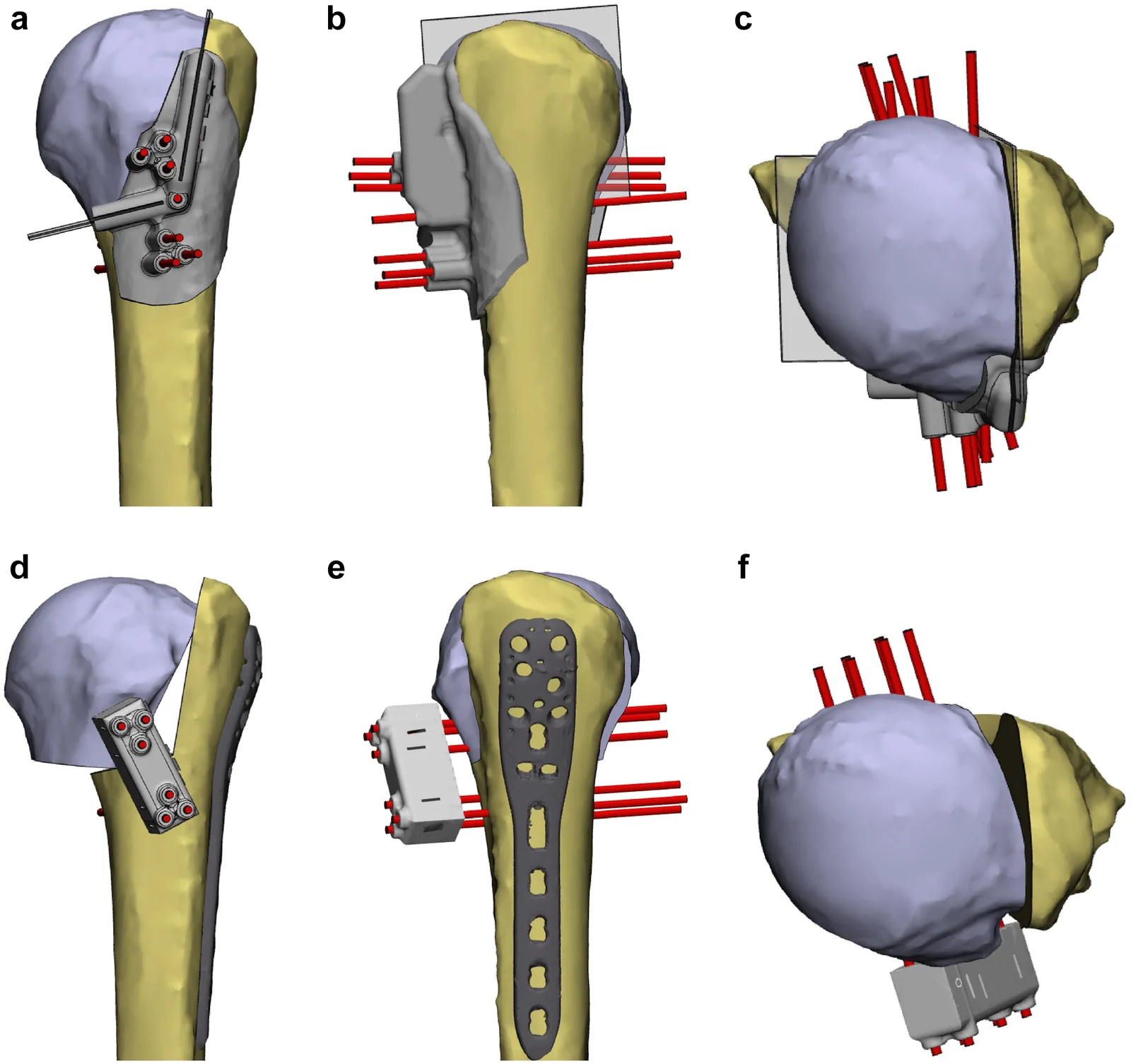
Three-dimensional planned corrective osteotomy according to the “humeral head-tuberosity” method. (**a** and **b**) The patient-specific cutting guide, aligned to the humerus with K-wires, for an osteotomy through the surgical neck and greater tuberosity. (**d** and **e**) The patient-specific reduction guide, guided by K-wires, with a PHILOS plate to fixate the osteotomized proximal humerus. (**c** and **f**) Cranial views to illustrate the rotational difference.

**Figure 5:**
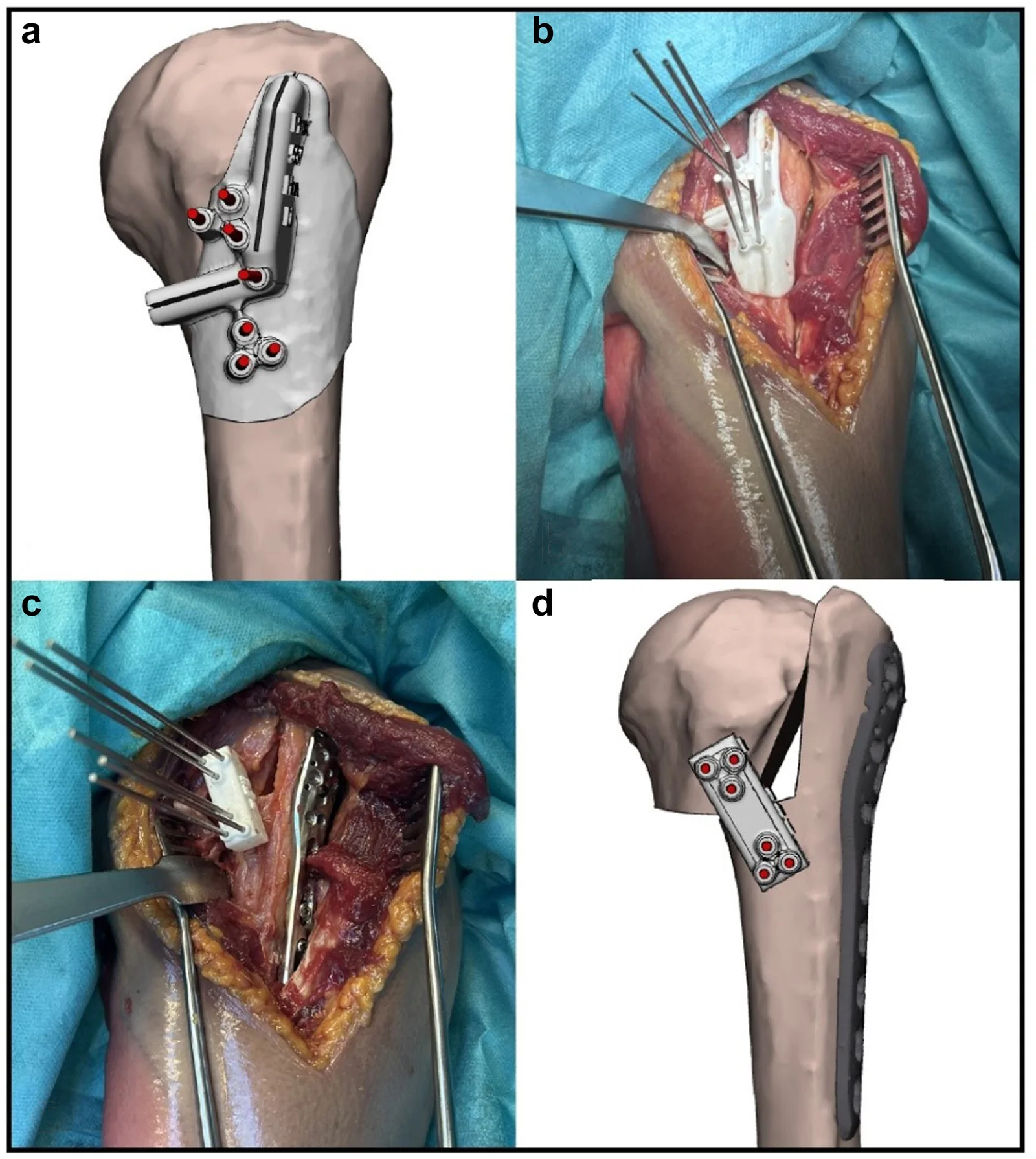
Visual representation of the “humeral head-tuberosity” procedure. (**a** and **b**) The 3D re-constructed patient-specific cutting guide and the intra-operative example, respectively. (**c** and **d**) The intra-operative example and 3D re-constructed patient-specific reposition guide with the PHILOS plate for fixation.

The second technique comprised the “humeral head-tuberosity separation” corrective osteotomy where an osteotomy through the humeral surgical neck and between the humeral head and GT was performed, using a patient-specific cutting and reduction guide and K-wires (Fig. 4). A deltopectoral approach was used for the anterior proximal humerus. After the preparation of the proximal humerus, the cutting guide (Figs. 4, *A-C* and 5, *A* and *B*) was placed on the bone and secured using K-wires (Figs. 4, *A-C* and 5, *A* and *B*). Two cuts were then performed using a cutting saw (max 0.6 mm thick), resulting in a V-shaped cut. Subsequently, the cutting guide was removed, and the reposition guide was placed to manipulate the fragments in the intended position, achieving the desired angulation and rotation (Figs. 4, *D-F* and 5, *C* and *D*). Then, the construct was fixated with a PHILOS plate (Synthes) in the new position (Figs. 4, *D-F* and 5, *C* and *D*). Finally, the reposition guide was removed.

### Post-operative evaluation

**Figure 6:**
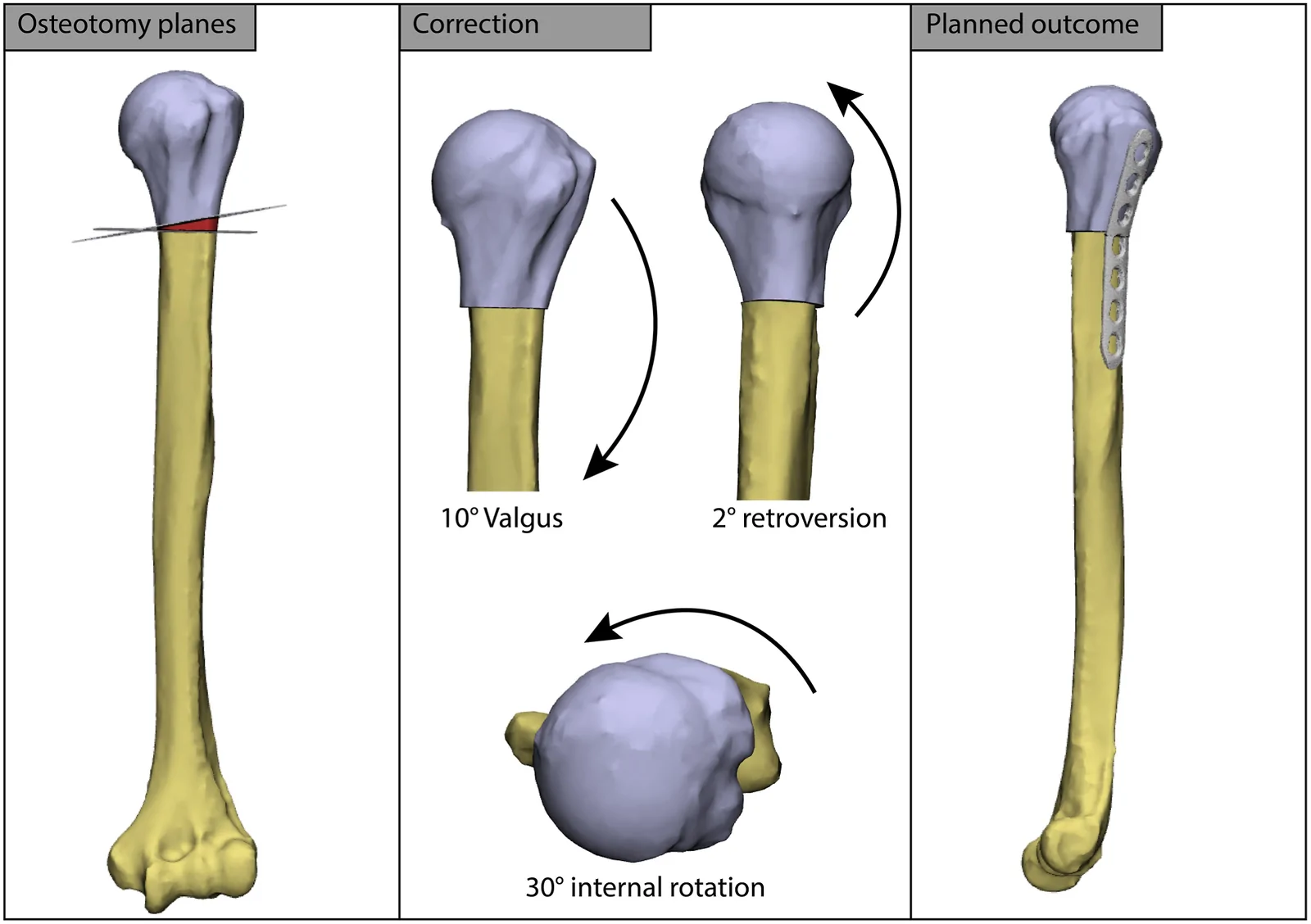
The planning process of an osteotomy. The osteotomy planes are determined, and subsequently, the predefined valgus and internal rotation degrees are applied. The result is the pre-operatively planned outcome of the correction.

For each human specimen, a post-operative CT scan was obtained with iterative metal artifact reduction techniques to assess corrections. A post-procedural 3D model of the (reversed) corrected humerus was created for all specimens using the Mimics Medical software. Subsequently, the virtually planned humerus fragment position was aligned with the post-operative position. Pre-operative, planned, and post-operative bone models were then compared in the 3-Matic software. Measurements were performed in a standardized coordination system for all humeri based on the anatomic planes. The rotational differences were measured between the fragments in the axial plane, and valgus-varus differences were measured in the coronal plane (Fig. 6). The mean translational error was used to quantify the overall displacement of the post-operative fragment relative to the planned position. The root mean square error (RMSE) was used to assess the surface deviation between the planned and post-operative fragments, defined as the average distance between corresponding points on the 2 surfaces of the 3D objects, with larger deviations weighted more strongly.

### Statistical analysis

Continuous variables are presented with medians and minimum and maximum values. Accuracy was evaluated for both techniques by assessing differences in degrees of rotation and angulation between the virtually planned 3D correction and the obtained corrections after surgery for each osteotomized humerus. Given the exploratory cadaveric design and small sample size, no inferential comparisons were undertaken. Inter- and intra-observer reliabilities of the post-operative 3D measurements were assessed by calculating the intra-class correlation coefficient, using a two-way mixed-effects model with absolute agreement and 95% confidence intervals. A statistical analysis was performed using R Statistical Software (v4.1.2; R Core Team 2021, Vienna, Austria).

## Results

Seven humeri underwent a corrective osteotomy with 3D patient-specific surgical guides. This resulted in an overall median difference between 3D pre-operative planning and post-operative results of 5.2° (min-max: 2.9°-8.5°) for rotation and an overall median difference of 3.4° (min-max: 2.4°-5.8°) for valgus angulation. Individual measurements for each specimen are shown in Table I.

**Table I:** Post-operative results of both 3D corrective osteotomy techniques.

Cases	Difference in rotation (°)	Difference in valgus angulation (°)	Mean translational error (mm)	Surface RMSE∗ (mm)
Extracapsular technique (planned 30° rotation, 10° valgus)				
Case 1	2.9	5.8	1.3	1.6
Case 2	5.2	3.2	2.3	2.6
Case 3	7.0	3.9	1.5	1.8
Median (range)	5.2 (2.9-7.0)	3.9 (3.2-5.8)	1.5 (1.3-2.3)	1.8 (1.6-2.6)
Humeral head-tuberosity separation technique (planned 10° rotation, 20° valgus)				
Case 1	3.6	3.6	1.2	1.4
Case 2	8.5	2.8	1.8	2.3
Case 3	8.3	2.4	1.2	1.5
Case 4	4.3	3.4	1.8	2.1
Median (range)	6.3 (3.6-8.5)	3.1 (2.4-3.6)	1.5 (1.2-1.8)	1.8 (1.4-2.3)

∗ Root mean square error (RMSE): Average deviation between corresponding points on the surfaces of the post-operative and planned fragments, with larger deviations weighted more strongly.

### Accuracy of extracapsular osteotomy

**Figure 7:**
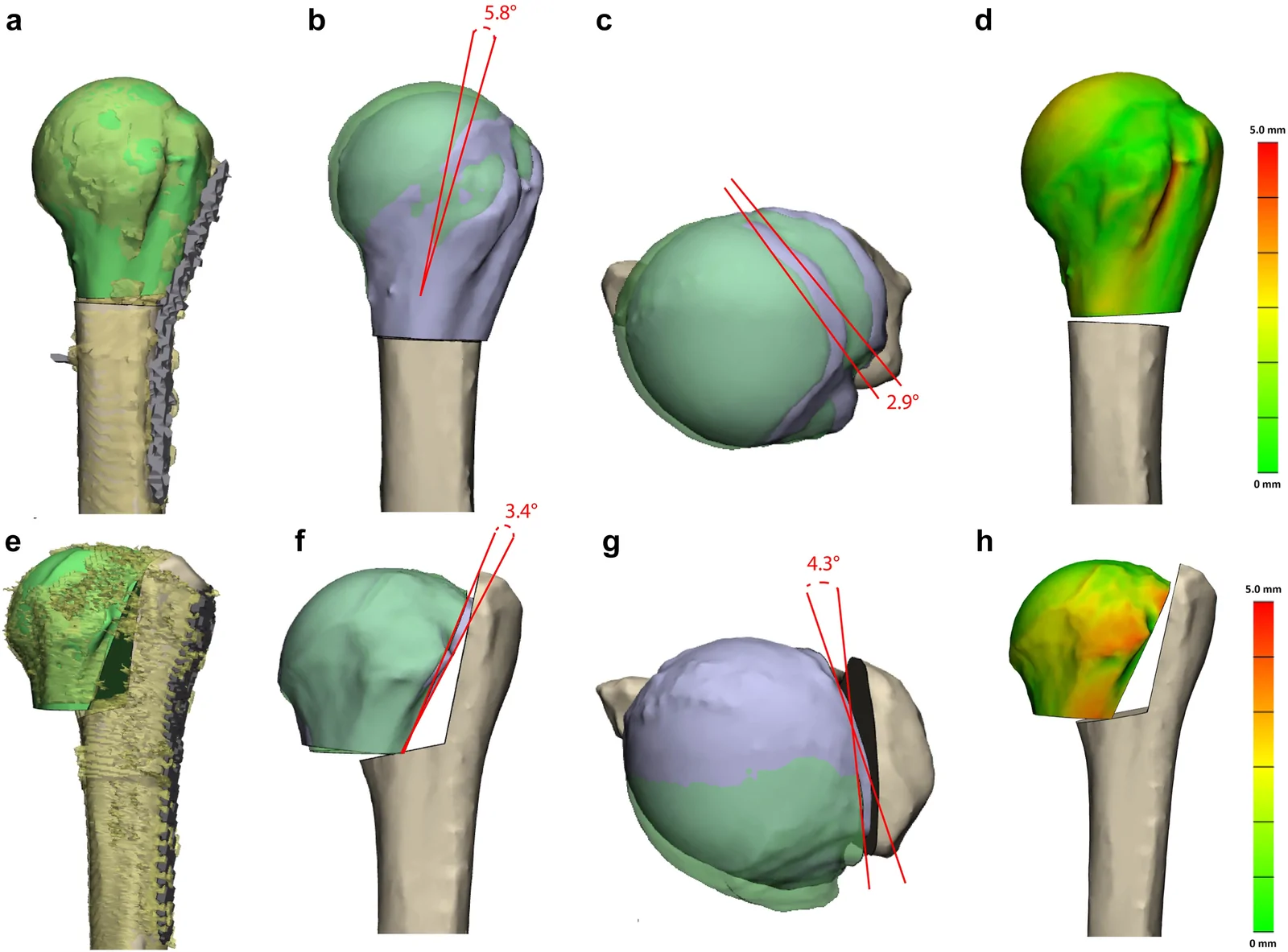
Evaluation of the planned corrective osteotomy and the achieved post-operative correction. (**a** and **e**) The fragments (*green*) from the 3D planning were aligned with the post-operative reconstruction for comparison. (**b** and **c**) The evaluation of the planned and achieved valgus angulation and rotation for the “extracapsular” osteotomy, with differences of 5.8° and 2.9°, respectively. (**f** and **g**) The evaluation of the planned and achieved valgus angulation and rotation for the “humeral head-tuberosity separation” osteotomy, with differences of 3.4° and 4.3°, respectively. (**d** and **h**) Post-operative surface deviations from the planned correction were quantified and visualized using heat maps.

For extracapsular osteotomies, 3 specimens underwent a “reversed” corrective osteotomy with unique patient-specific surgical guides. The median difference between the pre-operative planning and post-operative result in rotation was 5.2° (min-max: 2.9°-7.0°), and the median difference in valgus angulation was 3.9° (min-max: 3.2°-5.8°) (Table I). The median translational error was 1.5 mm (min-max: 1.3-2.3 mm), and the median RMSE was 1.8 mm (min-max: 1.6-2.6 mm). A visual representation of the surgical procedure and evaluation of a pre-operatively planned correction and the post-operative result for the extracapsular osteotomy of one of this study’s specimens are provided in Figs. 4 and 7, *A*-*D*.

#### Accuracy of humeral head-tuberosity separation osteotomy

Four specimens underwent a “reversed” corrective osteotomy with the humeral head-tuberosity separation technique. The median difference in rotation between the pre-operative planning and post-operative result was 6.3° (min-max: 3.6°-8.5°). For valgus angulation, the difference was 3.1° (min-max: 2.4°-3.6°) (Table I). The median translational error was 1.5 mm (min-max: 1.2-1.8 mm), and the median RMSE was 1.8 mm (min-max: 1.4-2.3 mm). A visual representation of the surgical procedure and evaluation of a pre-operatively planned and post-operative case are shown in Figs. 5 and 7, *E*-*G*, respectively.

#### Reliability

Repeated measurements performed by 2 independent observers demonstrated excellent inter-observer reliability for the post-operative angular measurements, with intra-class correlation coefficients of 0.90 and 0.87, respectively.

## Discussion

Corrective osteotomies for malunited PHFs are generally planned using 2D CT scans and performed with free-hand intra-operative techniques, possibly leading to less-predictable results. This feasibility study presents 2 innovative techniques using 3D patient-specific guides for corrective osteotomies to treat symptomatic proximal humeral malunions. Our study showed that both techniques are feasible and result in reconstructions with median deviations in rotation and angulation of 5.2° (min-max: 2.9°-8.5°) and 3.4° (min-max: 2.4°-5.8°), respectively. These deviations are in a similar range as the results in previous studies using 3D patient-specific guides for corrective osteotomies.^2^^,^^16^

To the best of our knowledge, this is the first study that measured in vitro accuracies of correction osteotomies using 3D patient-specific surgical guides for proximal humeral malunions. In both techniques, the median differences in rotation differed less than 7°, and the median differences in valgus angulation did not exceed 44°, indicating accurate executions of the corrective osteotomies. Previous literature has shown that shoulder biomechanics are altered at a varus deformity of 20°, leading to an increase of required elevation forces.^25^ Moreover, studies reported increased risk of glenohumeral joint degeneration when malrotation of the humeral head exceeds 20°.^8^^,^^12^ These thresholds for angular and rotational deformities are substantially higher than the reported post-operative deviations in this study. Therefore, the small deviations between the pre-operative plan and the achieved osteotomy are unlikely to affect clinical outcomes in future applications as they remain well within the range generally considered clinically acceptable for restoring proximal humeral anatomy. There is limited evidence available on the use of 3D patient-specific guides after malunited PHFs. Russo et al described the use of patient-specific guides for corrective osteotomies to prepare a varus malunited PHF for reverse total shoulder arthroplasty implantation^19^ and evaluated their method in a clinical study involving 20 patients with a 2-year follow-up period.^6^ However, the described technique was used to improve reverse shoulder prosthesis implantation in PHFs, which differs from our introduced techniques, aimed at restoring the anatomy. Moreover, post-operative accuracy was not assessed and therefore no comparison can be made between their studies and ours. Vlachopoulos et al provided a surgical strategy on pre-operative planning and designing patient-specific implants for a stepped approach using osteotomy and reduction guides to achieve the desired position of the proximal humerus.^24^ The guidelines for reduction are divided as a K-wire-based reduction (similar to our methods) or a reduction using a pre-drilled screw holes guide. This study showed that the two-step K-wire method^2^ is feasible in the shoulder through a deltopectoral approach. The second guide for reducing the bone fragments in the desired position, described in the guideline and used in this study, allows for accurate reduction and keeps the fragments in place during plate fixation. Moreover, we optimized the proposed designs of Vlachopoulos et al,^24^ performed the suggested methods in practice, and confirmed feasibility of the surgical approach in human specimens. Finally, we also provided 2 experimental techniques specifically for type 1D and type 4 deformities.

The first technique, or the “extracapsular” corrective osteotomy, is particularly useful in younger patients with PHF malunions where the anatomic relationship between the humeral head and GT remained intact, the type 1D deformities as described by Moineau et al.^15^ This osteotomy enables correction of malrotation in the axial plane and correction of varus deformities in the frontal plane. Ranalletta et al^18^ provided a case report of a successfully corrected PHF with a custom surgical guide. A wedge-osteotomy, similar to our extracapsular osteotomy technique, was performed to correct a varus malunited PHF, with satisfying functional outcomes after 28 months; the Constant score improved from 17 to 74.^18^ However, a patient-specific reduction guide was not used; this possibly results in inaccuracy as reduction is performed “free hand” and bone fragments may migrate during plate fixation. Moreover, contrary to our study, post-operative accuracy of the correction was not calculated.

The extracapsular osteotomy may not be sufficient for patients too young for arthroplasty with more severe malunited PHFs, where the anatomic relationships between the humeral head, GT, and surgical neck are disrupted (type 4 deformities).^5^ Therefore, we proposed a novel “humeral head-tuberosity separation osteotomy” in this study, which may offer a reproducible solution for these complex deformities. The method was proven feasible through a deltopectoral approach, with enough space for the cutting and reduction guides and little deviation from the pre-operative plan for correction of valgus angulation. Although soft-tissue structures such as the biceps and sub-scapularis tendon insertions require careful attention, this type of 3D-assisted corrective osteotomy remains feasible. Our proposed technique repositions the humeral head and lesser tuberosity relative to the GT and humeral shaft. Although all fragments are initially separated in type 4 fracture sequelae, alignment between the GT and shaft is often relatively well restored during the index surgery as these structures are generally easier to reduce and stabilize intra-operatively. Residual malalignment may therefore persist between the humeral head, LT, and GT-shaft complex. In such cases, our novel correction technique may potentially provide a solution without requiring complete separation of all fragments, particularly a GT osteotomy. Furthermore, 3D visualization is essential for accurate assessment in all planes, and patient-specific surgical guides eliminate potential inaccuracies because of visual estimation or “free-hand” reduction.^16^ Although the presented techniques are feasible and deviation in correction was little when compared to pre-operative planning, this study did not consider functional outcomes, costs, and planning/operating time. Future studies can compare the accuracy of 3D patient-specific surgical guides and conventional pre-operative planning with that of free-hand reconstruction for proximal humeral malunions. Moreover, future research could explore the use of patient-specific implants in combination with surgical guides to further improve accuracy. In addition, future studies should investigate the relationship of correction accuracy with clinical outcomes to determine clinically meaningful thresholds for residual deformity.

Corrective osteotomies can be a valuable solution for patients with malunited PHFs. In younger patients, it is an option to improve functional impairment without the need for arthroplasty. For older patients with type 4 malunions or type 1 malunions with a malunited GT, treatment with an osteotomy prevents arthroplasty implantation with a tuberosity osteotomy, the main predictor of poor outcomes.^5^^,^^7^ Nonetheless, it should also be noted that corrective osteotomies come with risks inherent to plate fixation, such as intra-articular screw cutout and avascular necrosis of the humeral head, especially in patients with osteoporosis.^23^ Moreover, functional outcomes may be unsatisfactory in case of damaged articular cartilage or rotator cuff deficiencies.

Several limitations should be considered when interpreting our findings. Because all specimens did not have any pre-existing bone deformities, an experimental design using reversed corrections had to be used to simulate the application of 3D patient-specific surgical guides for treating proximal humeral malunions, a setup proven feasible in previous in vitro research.^16^^,^^17^ Moreover, pre-defined degrees of correction were used, whereas pre-existing angular deformities in clinical practice vary considerably between patients. Although this design allows evaluation of the feasibility and accuracy of the patient-specific guides under controlled conditions, it does not fully replicate the clinical setting. Interestingly, this does not necessarily imply that clinical accuracy would be lower. In established malunions, callus formation and the resulting deviating bone morphology may provide additional anatomic reference points that facilitate more accurate positioning of the patient-specific guides. Furthermore, surrounding soft tissues have often adapted to the malunited position and tend to return to their anatomic alignment after correction, which may assist fragment reduction. In contrast, the cadaveric model required creation of an artificial deformity in an otherwise normal anatomy, effectively working against the native soft-tissue tension. Consequently, the accuracy observed in this cadaveric study may be representative of, or even slightly underestimate, the accuracy that can be achieved in clinical malunion cases. Nonetheless, clinical studies remain necessary to confirm these findings under real surgical conditions. Second, only 7 humeri were used in this study. Although this number was sufficient for this feasibility study, it was insufficient to perform a reliable comparison of potential differences in accuracy between the surgical techniques. A larger sample size is required to evaluate such differences. Third, this study did not compare corrections with 3D patient-specific guides to corrections using conventional planning. Nonetheless, using 3D patient-specific guides led to superior accuracies in other bone deformities,^14^ and this may be the case for PHFs as well. Fourth, our novel humeral head-tuberosity separation osteotomy may be useful in particular type 4 sequelae but does not completely mobilize the GT. It should be noted that the technique is not useful in cases that require mobilization of the GT. Theoretically, osteotomy through the humeral metaphysis could be extended to enable GT mobilization. We aim to explore this in a follow-up study. Fifth, we planned and performed correction osteotomies in the axial and coronal planes, as these represent the 2 most clinically important planes in humeral deformities. However, the sagittal plane was not considered in this study. Consequently, the presented techniques may not fully address the complexity of multi-planar malunions. Lastly, widespread clinical implementation of this workflow depends on the availability of the required resources and expertise. Although the presented methodology is not inherently dependent on a specific implant system or software platform, its application requires trained 3D planning personnel and access to a compliant printing facility. The planning process, including the design of the patient-specific templates, typically requires approximately 4-6 hours. Subsequently, production of the templates takes around 2 days, as the printed components require post-processing and sufficient cooling time before they are ready for clinical use. In addition, the printing costs are estimated to range between €100 and €200 per case. Therefore, broader applicability may depend more on institutional expertise, infrastructure, available production capacity, and associated costs and time than on the specific software or implant system used. Further research is needed to identify which proximal humeral fracture sequelae benefit most from this approach and to establish its clinical utility.

## Conclusion

Three-dimensional surgical planning and patient-specific surgical guides are feasible. The presented methods result in reconstructions with less than 10° deviation from pre-operative planning, demonstrating acceptable technical accuracy and supporting the potential of this approach for future clinical application. The extracapsular corrective osteotomy may serve as a solution for malunions with an intact anatomic relationship between the humeral head and GT. Moreover, the novel humeral head-tuberosity separation osteotomy may resolve challenging reconstructions of severe malunions where anatomy between the humeral head, GT, and surgical neck is disrupted. Future studies need to evaluate the proposed techniques in patients with actual proximal humeral malunions to assess post-operative accuracies and functional outcomes of the shoulder.

## Disclaimers:

Funding: No funding was received to conduct this study.

Conflicts of interest: All authors certify that there are no funding or commercial associations (consultancies, stock ownership, equity interest, patent/licensing arrangements, etc.) that might pose a conflict of interest in connection with the submitted article related to the author or any immediate family members.
